# Sorghum utilization in grain-based food products in China and Australia

**DOI:** 10.1371/journal.pone.0349203

**Published:** 2026-05-12

**Authors:** Anita Stefoska-Needham, Sophie L. Marsano, Liyan Zhong, Thomas H. Roberts

**Affiliations:** 1 School of Medical, Indigenous and Health Sciences, Faculty of Science, Medicine and Health, University of Wollongong, New South Wales, Australia; 2 School of Health Sciences, Faculty of Medicine and Health, University of New South Wales, New South Wales, Australia; 3 School of Life and Environmental Sciences, Faculty of Science, University of Sydney, Camperdown, New South Wales, Australia; 4 Sydney Institute of Agriculture, University of Sydney, New South Wales, Australia; University of Georgia, UNITED STATES OF AMERICA

## Abstract

Sorghum consumption has potential health-promoting effects for consumers. This study identified sorghum-containing grain-based food products available in major supermarkets in China and Australia. A total of 1,692 products were audited in Shenzhen, China and Illawarra, Australia, in 2023/24. Breakfast cereals and snack bars were evaluated in both countries, while flours, pastas, and noodles were evaluated only in Australia. Information on ingredients, including the presence of sorghum, food format, brand, product name, wholegrain/gluten-free labelling was recorded. In China, sorghum was found in 4.3% (12/279) of breakfast cereals, with only 1/12 sorghum-containing breakfast cereals listed sorghum in the first position of the ingredient list. Sorghum was found in 2.0% (9/458) of snack bars and was listed as either ‘sorghum’ (n = 3) or ‘sorghum flour’ (n = 6). In Australia, sorghum was found in 22/356 (6.2%) breakfast cereals, 9/285 (3.2%) snack bars, and was absent from all flours, pastas, and noodles. Most sorghum-containing cereals were extruded (36.4%) and labelled gluten-free (16/22, 73%) or wholegrain (14/22, 64%). Sorghum-containing snack bars, notably oat-bake and muesli bars, were mostly made from sorghum flour and flakes. Sorghum appeared in the first position in the ingredient list in 2/22 (9.1%) of breakfast cereals, and in the third or higher position for all snack bars. Among the breakfast cereal and snack bar subcategories analyzed, there were no significant differences in sorghum utilization between China and Australia (Fisher’s Exact Tests, p < 0.05), except for oat bake snack bars (higher in China, p = 0.0265). Overall, the audit data suggests that sorghum is not widely incorporated as an ingredient in common grain-based food products available to consumers in major Chinese and Australian supermarkets. Greater awareness of its potential consumer health benefits is needed to drive utilization of sorghum grain in foods across different markets.

## 1. Introduction

Sorghum (*Sorghum bicolor* (L.) Moench) is a gluten-free, cereal crop cultivated globally. In regions of the world such as north-west and south-central Africa, sorghum grain is traditionally consumed in staple foods, whereas in Western countries it is typically used in animal feed for domestic use or export, and to a much lesser extent in human foods [1]. The ability to grow sorghum in rain/temperature-variable climates, even in regions experiencing significant water scarcity, exemplifies sorghum’s potential to contribute to sustainable agriculture and food security around the world [2].

Nutritionally, sorghum is important due to its association with disease-mitigating mechanisms, notably those underpinning the development of cardiovascular disease, diabetes mellitus and some types of cancer [3,4]. The current evidence base, as appraised in a recent systematic literature review [5], indicates improvements in markers of oxidative stress, control of blood glucose and lipid levels. These benefits, along with enhanced satiety and appetite regulation relevant to weight management, appear particularly evident when wholegrain sorghum foods are consumed regularly. Experimental animal and cell-line research also provides evidence for favourable cell-mediated immune responses, including antioxidant and anti-inflammatory effects, particularly for polyphenol-rich red and brown sorghum grain [6]. Regular consumption of sorghum grain is needed for consumers to gain its purported health benefits. However, this can be difficult to achieve in societies where sorghum is not consumed traditionally, such as in Australia and China, and the sorghum-containing product range is limited [7,8]. Despite relatively limited utilisation, sorghum production in both Australia and China operates at a material and economically significant scale. Between 2020 and 2024, Australian sorghum production ranged from 397 thousand tonnes (kT) in a drought year (2020) to 2,648 kT in 2022, corresponding to annual values of US $105–704 million. Over the same period, China’s production remained relatively stable at 2,954–3,379 kT, with annual values of US $924–1,116 million (FAOSTAT, https://www.fao.org/faostat).

In countries with a limited range of sorghum-containing food products, more investment in food innovation is required to increase the volume and variety of sorghum-containing food products available to consumers. This must be done with consideration of consumers’ expectations of what constitutes healthy cereal-based foods. Consumer research indicates that the perceived healthiness of cereal foods, which is likely to drive consumer purchases, is associated with greater wholegrain, dietary fibre, and antioxidant contents, as well as lower levels of salt and fat [9]. From a nutrition science perspective, the beneficial, synergistic effects of the germ and bran fractions of wholegrains are typically implicated in health benefits associated with cereals, given they are high in dietary fibre, healthy lipids, micronutrients, and phytochemicals [10–12]. Sorghum-based food products can be made to possess these characteristics, and hence to be marketed as ‘healthy’, as well as ‘gluten-free’; however, attention must be given to the impact of processing methods on the health-enhancing potential and sensory appeal of end-products [13,14]. Monitoring progress towards greater sorghum-based product expansion and food innovation in Western societies requires regular evaluation of the prevalence of sorghum in products available to consumers, particularly within widely consumed supermarket product categories. As current monitoring largely focuses on production and trade metrics, food product audits are critical and provide an effective indicator of market uptake and dietary exposure [15,16]. Breakfast cereals and grain‑based snack bars are particularly relevant categories for this purpose due to their high population reach, routine consumption, and platforms for food innovation using alternative grains [17], and supermarkets serve as the dominant retail channel for these products. A world-first (to our knowledge) study of sorghum prevalence was conducted in 2020 through a cross-sectional audit of Australian supermarkets [18]. Sorghum ingredients were found in only 6.1% of ready-to-eat breakfast cereals and 2.0% of snack bars. The audit was limited to these two food categories in major supermarkets, and excluded smaller, specialty food retail outlets, posing a limitation given that innovative foods with lesser-known ingredients such as sorghum are more likely to be sold in such stores [18]. The 2020 audit provided important baseline data, with the authors recommending expansion to other categories in future audits to measure the prevalence in a wider range of food products. To date, no systematic supermarket audit has been conducted in China. Although sorghum grain is reported to be present in an expanding range of food products in Chinese supermarkets, including bread and noodles [19], this has not been empirically substantiated. Investigating the applications of sorghum in Chinese food products could offer valuable insights into how sorghum grain is utilized in this market and help drive demand for sorghum, supported by sorghum growers and the food industry globally.

The main aim of the present study was to examine the prevalence of sorghum in a range of grain-based food products in a cross-section of supermarkets in China and Australia. A secondary aim was to compare trends observed in the present audits to the previously mentioned, seminal Australian audit conducted in 2020.

## 2. Materials and methods

### 2.1 Overview of research methodology

**Fig 1:**
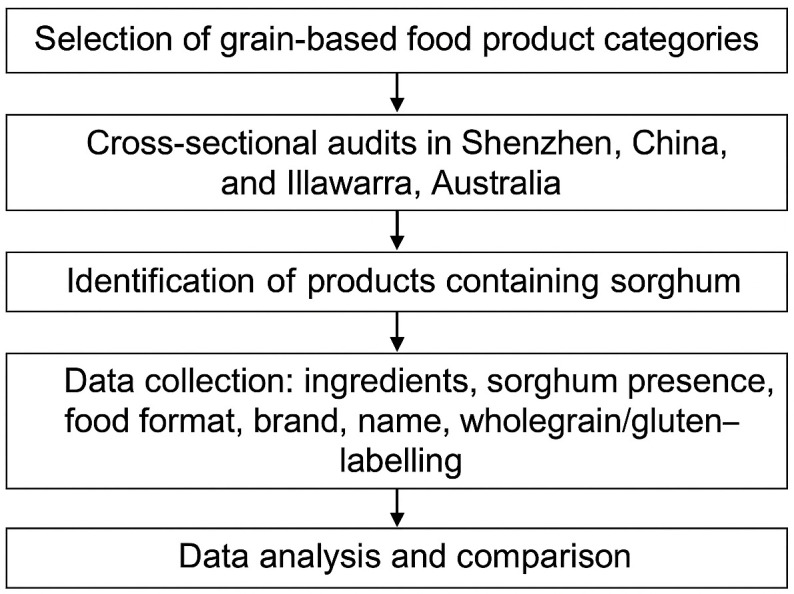
Research methodology used in this study.

The research methodology used in this study is represented schematically in Fig 1. Key stages of in this workflow are (1) selection of the grain-based food product categories, (2) the audits in China and Australia, (3) the identification of sorghum-based products, (4) data collection from the product labels, and (5) the analysis of the data and comparisons between datasets. Completion of all stages of the workflow allowed both the main aim and the secondary aim of the study (see previous section) to be achieved. The methodology adhered to standards that allow for research reproducibility, allowing valid comparisons to future studies.

### 2.2 Location of study and supermarket selection

Supermarkets were selected to maximize coverage of sorghum‑containing products within high‑volume retail settings rather than to represent shopper demographics. In China (Shenzhen), supermarkets were chosen based on researcher access to popular, large‑format stores (Bravo, Vanguard, and Walmart) with extensive packaged food ranges. In Australia (Illawarra, NSW), supermarkets were selected to reflect major chains by market share (Woolworths 38.2%, Coles 29.0% and Aldi 8.6%) and to span locations serving different socioeconomic status (SES) – Unanderra (low SES), Wollongong (medium SES) and Figtree (high SES). Health‑food specialty stores were also approached in the Australian audit to capture niche products that may appear outside mainstream channels. Permission from store managers was received either verbally or via email prior to conducting the audits in both China and Australia. No formal consent forms were required or requested by the stores, as the audit involved only observation and photography of products available for public sale, without any interaction with staff or customers.

### 2.3 Product selection

Product categories were selected on the basis that they contained grain-based foods. In China, breakfast cereals and snack bars were selected for analysis, as per the 2020 audit conducted in Australia [18]. Time constraints did not make it feasible to extend the audit beyond these two categories. In the present Australian-based audit, breakfast cereals and snack bars were also included, along with flour, pasta and noodle products, as these categories represent additional potential sources of sorghum in the Australian food supply. Definitions for all breakfast cereal subcategories applied in the classification and analysis are presented in Table 1, while definitions for snack bars are provided in Table 2.

**Table 1:** Definitions of breakfast cereal subcategories.

Subcategory	Definition
Biscuit	Ready‑to‑eat cereal products formed by compressing flakes or grain‑based mixtures into firm, biscuit‑shaped pieces. Typically baked and consumed as individual solid biscuits rather than loose cereal pieces.
Bran sticks	Extruded cereals made primarily from wheat bran and formed into elongated stick‑like pieces. High in dietary fibre and retain a rigid, rod‑like structure.
Bubbles/puffs/flakes	Ready‑to‑eat cereals produced by puffing, flaking, or expanding grains into loose, free‑flowing pieces (e.g., flakes, puffs, bubbles). Not compressed into solid forms.
Cluster	Cereals composed of small, aggregated clusters of grains, formed by binding flakes, oats, nuts, or seeds together using syrups or sweeteners, then baked to create a crunchy texture.
Combination cereal products	Products that combine more than one cereal format within the same package (e.g., flakes mixed with puffs, or clusters combined with extruded shapes) to create varied textures.
Extruded shapes	Cereals produced by forcing grain dough through an extruder to create uniform shapes (e.g., rings, stars, balls), often puffed or toasted to achieve the final texture.
Filled	Extruded cereal pieces with a soft or semi‑solid centre (e.g., chocolate, fruit paste, flavoured cream), encased in a crunchy outer shell.
Flake biscuits	Breakfast cereals in which flakes are compressed or moulded into cohesive biscuit‑shaped portions and baked, forming solid units rather than loose flakes.
Granola	Ready‑to‑eat cereals made from grains (commonly oats) mixed with oils and sweeteners, then baked or toasted to form crunchy clusters. Often includes nuts, seeds, or dried fruit.
Muesli	A loose mixture of raw or minimally processed grain flakes (often oats), nuts, seeds, and dried fruit. Not baked or toasted; typically eaten with milk, yoghurt, or soaked before consumption.
Oats	Products consisting of minimally processed oat grains, including rolled oats, quick oats, or steel‑cut oats. Typically require cooking or soaking and are used to prepare hot cereals such as porridge.
Porridge	A hot cereal made from oats or other grains cooked in water or milk to achieve a soft, semi‑solid consistency. Products usually require heating by the consumer.

**Table 2:** Definitions of snack bar subcategories.

Subcategory	Definition
Biscuit	Bars with a baked, biscuit-like base or texture, typically crisp and crumbly.
Cake	Snack bars with a soft, cake-like texture, often moist and sponge-based.
Cereal bar	Bars primarily composed of cereal grains bound together with sweeteners or syrups.
Filled	Bars containing a soft centre such as fruit paste, chocolate, or cream enclosed by a cereal or dough outer layer.
Muesli	Bars made from a mixture of oats, dried fruit, nuts, and seeds, typically minimally bound.
Nut	Bars where nuts are the primary ingredient, either whole or chopped and bound together.
Oat bake	Bars baked from an oat-based mixture, typically dense and soft, resembling baked oat slices.
Oat	Bars where oats are the dominant ingredient but not necessarily baked, including pressed or cold-formed bars.
Popcorn	Bars incorporating popcorn pieces bound together, often sweetened or flavoured.
Pressed	Bars formed through mechanical pressing of ingredients without baking, producing a dense texture.
Protein	Bars formulated with added protein isolates or concentrates as a key component.
Protein muesli	Bars combining muesli ingredients with added protein sources.
Protein nut	Bars combining nuts as primary ingredients with added protein sources.
Puff/ bubble	Bars incorporating puffed or expanded grains for a light, aerated texture.
Seed	Bars where seeds (e.g., pumpkin, sesame, sunflower) are the main ingredient.

### 2.4 Data collection

In China, data were collected over one week in February 2023. Eligible products were located in the conventional breakfast cereal and snack bar sections of all three supermarkets. The specialty sugar-free health food sections (commonly found in Chinese supermarkets) were also scanned for breakfast cereal products. In Australia, data collection took place over 3 weeks in April 2024. Eligible products were located in designated supermarket aisles for each category. Breakfast and snack bar products in the health food aisles were also assessed, given that grain-based food products increasingly dominate these locations in Australian supermarkets.

Data collection involved photographing the front-of-package, nutrition information panel (NIP), and ingredients list of each product. To avoid duplication, only products in the largest package size were recorded. If a product was sold at multiple store locations, it was counted only once. As per the methods reported previously [18], the following details were recorded for each product according to its product category: brand with product name, store location, product type (specific to each category), and presence or absence of sorghum in the ingredient list. The ingredients were only recorded for products in which sorghum was identified, including sorghum’s position in the ingredients list. In both China and Australia, ingredients must be listed in descending order by in-going weight, meaning the first ingredient listed contributes to the largest weight in the product and the last ingredient contributes to the least weight. Ingredient lists were therefore used in this study as a semi‑quantitative indicator of composition; however, in the absence of percentage declarations, the actual proportion of sorghum could not be reported and no inference about dosage could be made from rank alone. The following product details were also recorded: percentage contribution of sorghum (if available), sorghum format (e.g., wholegrain, flaked or puffed), any health/ nutrition related claims or food attributes (if present, e.g., gluten free), target audience (if stated, e.g., children). In the Australian audit, a note was made if the same product was included in the 2020 audit.

### 2.5 Data analysis

Descriptive statistics were used to analyze the data using Microsoft Excel 365 (version 16.50). Counts and percentages were used to evaluate the prevalence of sorghum in different product categories (breakfast cereals, snack bars in both China and Australia; flours, pastas and noodles in Australia only) and their subcategories, which were based on sorghum format, e.g., flaked or puffed. Where relevant, findings were compared with the 2020 Australian audit [18] to contextualise changes observed between the two time points. An in-depth trend analysis was not conducted.

To enable a statistical analysis of sorghum prevalence in supermarkets in China and Australia, various product subcategories were combined to reduce the total number of subcategories. The counts of sorghum-containing products were too low to allow valid Chi-square tests to be performed; therefore, a Fisher’s Exact Test was performed on each contingency table (2 × 2), using GraphPad Prism v10.5.0. A Fisher’s Exact Test was also performed on the total breakfast cereal counts and total snack bar counts, again comparing China to Australia.

### 2.6 Inclusivity in global research

Additional information regarding the ethical, cultural, and scientific considerations specific to inclusivity in global research is included in the Supporting Information (S1 Checklist).

## 3. Results

In this study, a total of 1,692 products were evaluated across five different grain-based product categories in China and Australia. In China, 737 food product items were analyzed. Of these, 279 items were categorized as breakfast cereals, while the remaining 458 items were categorized as snacks. In Australia, a total of 955 products were audited, and of these, 356 were breakfast cereals, 285 were snack bars, 80 were flours, 59 were noodles, and the remaining 175 products were pastas.

### 3.1 Breakfast cereal audit in China

Of the total 279 breakfast cereal products audited in China, porridge-style cereals were most frequently identified (n = 93, 33.3%), followed by bubbles/puffs/flakes (n = 76, 27.2%), and muesli (n = 69, 24.7%) (Table 3). Flaked biscuits and granola comprised the remaining cereal product types (10%). Only 12 (4.3%) of the 279 cereals examined, contained sorghum across a range of breakfast cereal types, including porridge (n = 5), bubbles, flakes and puffs (n = 3), muesli (n = 3), and flaked biscuits (n = 1) (Table 3).

**Table 3:** Summary of sorghum-containing breakfast cereals in major supermarkets in Shenzhen, China (February 2023), and major supermarkets and a smaller health food store in the Illawarra region of New South Wales, Australia (April 2024). Data are presented as counts (n) and percentages (%).

Product subcategory description	Products surveyed in each subcategory (n)	Products in each subcategory as a percentage of total surveyed products (%)	Sorghum-containing products in each subcategory (n)	Sorghum-containing products per subcategory as a percentage (%)	Sorghum-containing products per subcategory as a percentage of total surveyed products (%)
**China**					
Bubbles/puffs/flakes	76	27.2	3	3.9	1.1
Flake biscuits	13	4.7	1	7.7	0.3
Muesli	69	24.7	3	4.4	1.1
Granola	28	10.0	0	0.0	0.0
Porridge	93	33.3	5	5.4	1.8
**Total**	**279**	**100.0**	**12**		**4.3**
**Australia**					
Biscuit	17	4.8	5	29.4	1.4
Bran sticks	5	1.4	0	0.0	0.0
Cluster	39	11.0	0	0.0	0.0
Combination cereal products	21	5.9	1	4.8	0.3
Extruded shapes	39	11.0	8	20.5	2.2
Filled	1	0.3	0	0.0	0.0
Granola	51	14.3	3	5.9	0.8
Muesli	47	13.2	2	4.3	0.6
Oats	34	9.5	0	0.0	0.0
Porridge	45	12.6	2	4.4	0.6
Puffs/bubbles/flakes	57	16.0	1	1.8	0.2
**Total**	**356**	**100.0**	**22**		**6.2**

Of the sorghum-containing breakfast cereals identified, only 1/12 listed sorghum in the first position of the ingredient list. Sorghum was listed second in the ingredient list in 2/12 of the products, and in the third position or beyond in 9/12 products. The most common sorghum ingredient in the ingredient list was sorghum flour (5/12), followed by wholegrain sorghum (3/12), sorghum (3/12), and organic sorghum (1/12). Eight of the 12 (66.7%) sorghum-containing breakfast cereal products had wholegrain sorghum listed as an ingredient, while the remaining products did not specify whether the grain/flour was wholegrain. As a percentage of the total surveyed breakfast cereals, sorghum was most frequently used in porridge (1.8%), followed by muesli and bubbles/puffs/flakes (1.1%, respectively) (Table 3). A total of seven out of 12 sorghum-containing breakfast cereals were gluten-free, and three out of 12 were marketed towards children and were made with wholegrain sorghum. Most of the sorghum-containing breakfast cereals (9/12) were in the health food section of the supermarket. Only three out of 12 of the sorghum-containing breakfast cereals mentioned ‘sorghum’ on the front-of-pack label, with the remainder mentioning ‘sorghum’ only in the list of ingredients or in small font on the back of the pack. All 12 sorghum-containing breakfast cereals included additional descriptors such as ‘organic sorghum’, ‘sorghum rice’, ‘sorghum flour’, or ‘puffed sorghum’.

### 3.2 Breakfast cereal audit in Australia

Breakfast cereals audited in Australia comprised 12 subcategories: biscuit, bran sticks, cluster, combination cereal products, extruded shapes, filled, flakes, granola, muesli, oats, porridge and puffs/bubbles (Table 3). Of these subcategories, granola was the most common (51/356, 14.3%), followed by muesli (47/365, 13.2%) and porridge (45/365, 12.6%). Of the total cereals assessed, only 22 of 356 (6.2%) contained sorghum as an ingredient. The top six formats of sorghum ingredients were extruded shaped cereals, followed by biscuit, granola muesli, porridge, combination cereal products and flakes. Sorghum was not identified in any breakfast cereal products in the specialty health food store audited.

Sorghum was found in extruded-shaped breakfast cereals more commonly (36.36%) compared to breakfast cereal subcategories (Table 3). Sorghum was positioned first in the ingredients list in 2/22 (9.1%) of sorghum-containing breakfast cereal products, listed second in 7/22 (31.8%) of products, third in 10/22 (45.5%) of products and greater than third in 3/22 (13.6%) of products. In these breakfast cereal products, sorghum was most commonly listed as sorghum flour (n = 13), followed by sorghum (n = 3), wholegrain sorghum flour, and sorghum.

Sixteen of the 22 products (73%) were gluten-free, two of which stated ‘gluten-free flour blend’ in the ingredients list, but further details relating to the type of sorghum flour were not provided. Children were the target market for 5/22 (23%) of these products, based on label designs including images. Only 4/22 (18%) of products spelled out the word ‘sorghum’ anywhere on the packaging outside of the ingredients list. Wholegrain claims were found on 14/22 (64%) of breakfast cereal products, and out of these products, 6/22 (27%) contained wholegrain sorghum in the ingredient list. All the products that contained wholegrain sorghum in the ingredients list made wholegrain claims. ‘Source of fibre’ was a popular claim with all products making a dietary fibre claim on the front-of-pack label.

### 3.3. Snack bar audit in China

A total of 458 snack products were analyzed in eight subcategories (Table 4). Only 2.0% (9/458) of the snack bar products contained sorghum as an ingredient. Of these, three were classified as biscuit bars (33%), two as muesli/cereal bars (22%), two as puff/bubble bars (22%), one as oat bake bars (11%), and one as a rolled-oat bar (11%).

**Table 4:** Summary of sorghum-containing snack bars in major supermarkets in Shenzhen, China (February 2023), and in major supermarkets and a smaller health food store in the Illawarra region of New South Wales, Australia (April 2024). Data are presented as counts (n) and percentages (%).

Product description/style	Products surveyed in each subcategory (n)	Products in each subcategory as apercentage of total surveyed products (%)	Sorghum-containing products in each subcategory (n)	Sorghum-containing products per subcategory expressed as a percentage (%)	Sorghum-containing products per subcategory as a percentage of total surveyed products (%)
**China**					
Biscuit	108	23.6	3	2.8	0.7
Cake	72	15.7	0	0.0	0.0
Muesli	58	12.7	2	3.4	0.4
Nut/seed	63	13.8	0	0.0	0.0
Oat bake	32	7.0	1	3.1	0.2
Pressed	28	6.1	0	0.0	0.0
Puff/bubble	12	2.6	2	16.7	0.4
Rolled oat	85	18.6	1	1.2	0.2
**Total**	**458**	**100.0**	**9**		**2.0**
**Australia**					
Biscuit	1	0.4	0	0.0	0.0
Cake	7	2.5	0	0.0	0.0
Cereal bar	10	3.5	0	0.0	0.0
Filled	19	6.7	0	0.0	0.0
Muesli	26	9.1	2	7.7	0.7
Nut	61	21.4	0	0.0	0.0
Oat bake	31	10.9	7	22.6	2.5
Oat	18	6.3	0	0.0	0.0
Popcorn	1	0.4	0	0.0	0.0
Pressed	59	20.7	0	0.0	0.0
Protein	14	4.9	0	0.0	0.0
Protein muesli	11	3.8	0	0.0	0.0
Protein nut	7	2.5	0	0.0	0.0
Puff/ bubble	17	5.9	0	0.0	0.0
Seed	3	1.0	0	0.0	0.0
**Total**	**285**	**100.0**	**9**		**3.2**

The utilization of sorghum varied across the different types of snack bars. In biscuit-style bars, muesli/cereal-style bars, and oat bake-style bars, sorghum was incorporated in formulations as ‘sorghum flour’ or ‘wholegrain sorghum’. Sorghum was identified in the 8^th^ or higher position in the ingredients list. However, in the other categories, ‘puffed sorghum’ was listed in the ≥ 5^th^ positions in the ingredients list, and the product descriptor included ‘puffed product’.

Food manufacturers did not specify whether or not wholegrain sorghum ingredients were used in the bars. All sorghum bars were labelled gluten-free, with 3/9 (33%) marketed toward infants and children. Although none of the snack bars mentioned sorghum in their product names, some displayed sorghum on the front-of-pack labels. Additionally, none of the sorghum-containing snack bars included the term ‘ancient grain(s)’ in their ingredient lists across all subcategories.

### 3.4 Snack bar audit in Australia

In Australia, the snack bar audit comprised 285 products across 15 subcategories (Table 4). These were biscuit style, cake bar, cereal bar, filled, muesli bar, nut bar, oat bake bar, oat bar, popcorn bar, pressed bar, protein bar, protein muesli bar, protein nut bar, puff/bubble, seed bar. Of these categories, nut bars were the most common (21.4%), followed by pressed bars (20.7%) and oat bake bars (10.9%). Of the audited bars, nine out of 285 (3.2%) contained sorghum as an ingredient (Table 4). Sorghum was not identified in any snack bar products in the specialty health food store audited.

The sorghum-containing snack bars comprised only two categories, oat bake bars (n = 7) and muesli bars (n = 2). Sorghum’s position in the ingredients list was greater than position three for all the snack bar products audited. Sorghum flour (n = 7) and sorghum flakes (n = 6) were the most common formats in the snack bars, followed by puffed sorghum (n = 2). No bars specifically identified that the sorghum utilized was wholegrain. Of the sorghum-containing snack bars audited, none were perceived to be specifically targeted at children. All the sorghum-containing snack bars were gluten-free. Two out of the nine (22%) sorghum-containing snack bars stated the word “sorghum” on the product packaging, as well as in the ingredients list.

### 3.5 Audit of pastas, noodles, and flour products in Australia

The audit of pastas, noodles, and flour products did not identify sorghum in any ingredient formulations, including generically labelled gluten-free options in these product categories.

### 3.6 Sorghum prevalence in breakfast cereals and snack bars in supermarkets in China and Australia

Among the breakfast cereal (Table 5) and snack bar (Table 6) subcategories analyzed, there were no significant differences in sorghum utilization between China and Australia (Fisher’s Exact Tests, p < 0.05), except for oat bake snack bars, of which one (of 31) were from China and seven (of 24) from Australia (p = 0.0265).

**Table 5:** Fisher’s exact tests of sorghum prevalence in breakfast cereals in supermarkets in China (February 2023) versus Australia (April 2024). Data are derived from Table 3.

Product subcategory description/style	Country	Sorghum-containing products in each subcategory	Non-sorghum-containing products in each subcategory	P value
Bubbles/puffs/flakes	China	3	73	0.6349
	Australia	1	56	
Biscuits/flake biscuits	China	1	12	0.1961
	Australia	5	12	
Muesli/granola	China	3	94	0.7208
	Australia	5	93	
Porridge	China	5	88	>0.9999
	Australia	2	43	
All others *	China	0	0	>0.9999
	Australia	9	130	
**Total**	China	**12**	**267**	0.3751
	Australia	**22**	**334**	

* *Includes all remaining breakfast cereal subcategories not itemised in this table; full definitions of these subcategories are provided in* Table 1.

**Table 6:** Fisher’s exact tests of sorghum prevalence in snack bars in supermarkets in China (February 2023) versus Australia (April 2024). Data are derived from Table 4. Contingency tables are 2 × 2. An asterisk highlights p values < 0.05.

Product subcategory description/style	Country	Sorghum-containing products in each subcategory	Non-sorghum-containing products in each subcategory	P value
Biscuits	China	3	105	>0.9999
	Australia	0	1	
Cake	China	0	72	>0.9999
	Australia	0	7	
Muesli/protein muesli	China	2	56	0.6414
	Australia	2	35	
Nut/protein nut/seed	China	0	63	>0.9999
	Australia	0	71	
Oat bake	China	1	31	0.0265*
	Australia	7	24	
Pressed	China	0	28	>0.9999
	Australia	0	59	
Puff/bubble	China	2	10	0.1626
	Australia	0	17	
Rolled oat/oat	China	1	84	>0.9999
	Australia	0	18	
All others *	China	0	0	>0.9999
	Australia	0	44	
**Total**	China	**9**	**449**	0.3322
	Australia	**9**	**276**	

* *Includes all remaining snack bar subcategories not itemized in this table; full definitions of these subcategories are provided in* Table 2.

## 4. Discussion

In this cross-sectional study, we investigated the utilization of sorghum in grain-based food products across food retail outlets in China and Australia. To our knowledge, this study represents the first benchmarking of sorghum’s prevalence in cereal-based food products in China, and builds upon prior research in Australia that included a seminal audit conducted in 2020 [18]. Overall, sorghum prevalence in Chinese breakfast cereals and snacks was similar to that observed in Australia, with sorghum identified in 8.3% of 279 breakfast cereals and 2.0% of 458 snack bars audited in China, compared to 6.2% of 356 breakfast cereals and 3.2% of 285 snack bars in Australia (refer to Table 3). The Australian findings represent a slight rise from 6.1% for breakfast cereals and 2.0% for snack bars in 2020 [18]. The review of pastas, noodles and flour products reflects an expanded audit undertaken exclusively within Australia and should not be interpreted as part of any cross-country comparisons. Notably sorghum was not identified in any formulations within these product categories.

We wish to emphasize that since the sorghum-containing products were rare (e.g., only 12 out of 279 breakfast cereals in China and 22 out of 356 in Australia), the sample size is very small, resulting in low statistical power and a high risk of type-II error (failure to detect a real difference). Therefore, in this study, a conclusion that no significant difference was found cannot be used confidently to imply that no difference exists.

In China, approximately one-third of sorghum-containing breakfast cereals were identified in the porridge subcategory (refer to Table 3). In Australia, sorghum was more commonly found in ready-to-eat extruded breakfast cereals (refer to Table 3). In China, porridge is often prepared using a powdered or paste base that is mixed with milk or water, offering a novel format with potential applicability in Australian markets. In these mixtures, sorghum is typically combined with other grains such as corn, barley, and red bean to create a porridge-type product. Whether this type of product could serve as a viable breakfast cereal alternative for Australian consumers requires further testing and validation by food manufacturers willing to explore new product developments.

The breakfast cereal markets in both China and Australia have experienced significant growth due to increasing consumer interest in healthy and nourishing ready-to-eat breakfast options. From 2012 to 2017, the market in China showed a yearly growth rate of 10.2% [20], and it is projected to reach a revenue of US$1.32 billion in 2024 [21]. This high demand presents an opportunity for commercial success in the breakfast cereal market by expanding the sorghum breakfast cereal product range in both countries. Targeting young people aged between 2 and 18 years old may be a key strategy for sorghum food innovators and research and development, given that 44.1% of all wholegrain products annually are consumed by this group in Australia [18,22]. In China, grain foods are staples in the diets of consumers across all age groups and typically include rice and wheat products (such as noodles and steamed buns), but the prevalence of sorghum in these commonly consumed foods is currently unknown. Noodles and pasta products were reviewed in the Australian audit presented in this paper, but sorghum was not identified in any formulations.

Compared with breakfast cereals, sorghum prevalence in snack bars was observed to be lower in both China and Australia (refer to Table 4), likely due to the significantly smaller snack bar market relative to the breakfast cereal market in these countries [23,24]. Snack bar formulations in China were largely comprised of sorghum flour, which is produced by milling sorghum grain to formulate different types of snack bar formats, including the more popular biscuit-style and rolled oat-style bars. These applications appear to be an extension of traditional Chinese foods, such as mantou, offering potential ideas for novel product innovation in this category in Australia.

Although sorghum flour was identified in the snack bar products in China, it was rarely the dominant or characterizing ingredient. In the biscuit-style snack bars, it typically comprised about 5% of the ingredients, alongside other grains like oats (≥ 5%) and corn (≥ 5%). In the Australian audit, nut bars were observed to still dominate since 2020, potentially explaining the low prevalence of sorghum in this category [18]. Like in China, the sample of sorghum-containing snack bars comprised mostly sorghum flour, followed by sorghum flakes then puffed sorghum. This contrasted with the 2020 audit, where sorghum flour was not listed in the ingredients list of any snack bars in Australia, and puffed sorghum was most common [18].

The marketing of sorghum in both breakfast cereals and snack bars is limited in both countries. For example, in Australia, only two of the snack bars included ‘sorghum’ on the packaging, and it was listed for its texture contribution rather than its health effects. The inclusion of sorghum on the front-of-packaging would be a step in the right direction to increase consumers’ awareness of sorghum, as lack of recognition has been identified as a major barrier to acceptance of sorghum as a human food [7].

In China, the healthier sorghum-containing snack bars, characterized by higher contents of wholegrain and/or fibre and low or no added sugars, tended to be marketed towards children and younger people. Examples of these products included wholegrain sticks and brown rice crackers. In contrast, in Australia, there were no sorghum-containing snack bar products targeted towards children, despite being a convenient and easy lunch box option. According to the earlier Australian audit [18], manufacturers of the grain-based snack bars may have little motivation to explore new formulation ideas, such as using sorghum alongside more fibre and less sugar, due to their strong market position with current offerings. However, demonstrating other food successes in other markets, such as in China, may stimulate ideas for new product development to increase market share in Australia. Furthermore, wholegrain sorghum was not listed in the ingredients of any snack bars containing sorghum in Australia, nor was there a wholegrain claim displayed, despite wholegrain claims generally being used more frequently on Australian food products [25]. Research by the Grains and Legumes Nutrition Council of Australia indicates that Australian consumers do not prioritize consumption of wholegrains, despite having an awareness of their potential health benefits [26], implying that marketers may not be interested in leveraging wholegrain claims to drive consumer purchases in the snack bar product category.

Differences in the availability and types of sorghum‑containing products may reflect underlying cultural and economic influences. In Australia, sorghum has a long history as a locally grown grain used predominantly in livestock feed, with recent growth in consumer‑oriented applications linked to health‑focused marketing and interest in gluten‑free products [7]. In contrast, Chinese grain consumption patterns are shaped by longstanding cultural preferences for rice, wheat‑based noodles, and traditional coarse grains such as millet, which may reduce consumer familiarity with sorghum as an ingredient [19]. These cultural and economic contexts likely contribute to differences in product development and category representation observed between the two markets. To support broader utilization across settings, cross‑cultural consumer education that raises awareness of sorghum’s culinary uses and nutritional attributes, led by chefs in restaurants, grain advocacy groups, and health professionals (notably dietitians and nutritionists), may be an effective strategy.

A cross-section of specialty health food retailers was intended to be included in the Australian audit presented here; however, only one store agreed to be involved. Despite this, important insights were afforded by this single site. Although sorghum was not found in any breakfast cereals, snack bars, flours, pastas or noodles, the diversity of product offerings reflected more specialized, health-focused products, particularly in the breakfast cereal and flour categories. These findings suggest potential for sorghum’s incorporation into novel formats. Expanding future audits to include more health food and specialty shops could provide a more comprehensive understanding of emerging trends. Including gym and fitness stores, which cater to health-conscious consumers, may further reveal opportunities for sorghum innovation. Also, future audits should focus on ethnic food specialty stores, especially to capture people from cultural backgrounds that use sorghum in traditional cooking, such as people from African and Indian communities. The bread category should also be audited in future across a variety of retail outlets, including supermarkets, specialty food stores and bakeries, as bread is the most frequently purchased grain food in Australia after refined pasta and rice [26].

Future opportunities for sorghum food innovation in both China and Australia should leverage the potential health benefits and food attributes associated with wholegrains broadly. Consumer preferences in both countries are shifting towards health-conscious and diet-focused choices, with many consumers willing to pay a premium for perceived health benefits [27,28]. The rising demand for gluten-free products has also opened opportunities for smaller producers, driving innovation and competition [29]. The healthy breakfast and snack food market will likely grow in response to increased demand for convenient, health-oriented products. Wholegrain foods, particularly those with a low glycemic index, have also gained popularity [30], presenting opportunities for expanding sorghum-based offerings. The growing interest in plant-based diets [31] also positions sorghum as a viable ingredient for meeting these evolving demands, potentially increasing its acceptance and prevalence in human food supplies. Targeting young consumers (up to 18 years) in both countries presents an opportunity to expand the sorghum breakfast and snack bar cereal market, as this demographic contributes significantly to wholegrain consumption [22]. In China, sorghum is most recognized for its use in liquor production; however, during the auditing of products for this study, sorghum was also identified in cooking sauces and snacks (results not reported). This highlights sorghum’s versatility and potential for diverse applications [19].

The key strengths of this study include the rigorous methodology and cross-country analysis, which provided valuable insights into sorghum’s market presence. The large sample size and standardized data collection methods strengthen the study’s reliability. However, between-country differences in food labelling regulations and dietary preferences, as well as the inability to specify sorghum content due to labelling practices, constrained direct comparisons. The focus on popular, large‑format supermarkets and major chains was intended to maximise product coverage but may underestimate sorghum presence in smaller independent stores, convenience outlets, e‑commerce platforms, or region‑specific retailers. Accordingly, cross‑setting comparisons were limited to contextualising differences rather than implying real‑time equivalence or national representativeness.

Temporal mismatch may also be perceived as a limitation. The China (February 2023) and Australia (April 2024) audits were conducted approximately one year apart; however, each audit was intentionally designed as a snapshot of the market at a specific point in time, rather than as synchronised assessments intended for real‑time cross‑country comparison. The two markets differ in retail structure, product turnover, cultural grain preferences, and the pace of new product development, and the study was not designed to evaluate tightly aligned market dynamics across settings. Accordingly, cross‑country observations are framed cautiously to contextualise differences rather than imply concurrent equivalence.

Limited access to health food and speciality shops in Australia also restricted the scope of the audit. The findings from Shenzhen may have limited generalisability to other regions of China, particularly northern and western provinces with different dietary patterns. Similarly, the Illawarra‑based Australian audit may not fully represent consumer purchasing environments elsewhere in Australia. Ethical considerations prevented the identification of specific brands or stores, and ingredient percentage data for sorghum was not always available, limiting the granularity of the analysis.

## 5. Conclusions

This study highlights a positive trend in sorghum’s integration into grain-based food products in China and Australia and identifies opportunities for further market penetration through innovation and education. Emphasizing sorghum’s health benefits and expanding its applications in novel and traditional formats can foster greater consumer acceptance and demand. Strategic product development, combined with targeted marketing focused on health benefits, can enhance sorghum’s appeal to consumers, fostering greater adoption in diverse food categories.

Future research should explore the nutritional and sensory benefits of sorghum-containing products and assess their appeal across diverse consumer groups. Expanding audits to additional food categories and evaluating new product formats could uncover further opportunities for sorghum innovation. Audits of specialty retailers, including those selling diverse ethnic foods as well as health food stores, would also provide a more comprehensive understanding of emerging trends and opportunities.

## Supporting information

S1 File Inclusivity in global research. (DOCX)
